# Land Use History Shifts *In Situ* Fungal and Bacterial Successions following Wheat Straw Input into the Soil

**DOI:** 10.1371/journal.pone.0130672

**Published:** 2015-06-23

**Authors:** Vincent Tardy, Abad Chabbi, Xavier Charrier, Christophe de Berranger, Tiffanie Reignier, Samuel Dequiedt, Céline Faivre-Primot, Sébastien Terrat, Lionel Ranjard, Pierre-Alain Maron

**Affiliations:** 1 INRA, UMR 1347 Agroecology, Dijon, France; 2 Centre de recherche Poitou-Charentes, INRA, Lusignan, France; 3 INRA, Plateforme GenoSol, UMR1347 Agroecology, Dijon, France; Netherlands Institute of Ecology (NIOO/KNAW), NETHERLANDS

## Abstract

Soil microbial communities undergo rapid shifts following modifications in environmental conditions. Although microbial diversity changes may alter soil functioning, the *in situ* temporal dynamics of microbial diversity is poorly documented. Here, we investigated the response of fungal and bacterial diversity to wheat straw input in a 12-months field experiment and explored whether this response depended on the soil management history (grassland vs. cropland). Seasonal climatic fluctuations had no effect on the diversity of soil communities. Contrastingly fungi and bacteria responded strongly to wheat regardless of the soil history. After straw incorporation, diversity decreased due to the temporary dominance of a subset of copiotrophic populations. While fungi responded as quickly as bacteria, the resilience of fungal diversity lasted much longer, indicating that the relative involvement of each community might change as decomposition progressed. Soil history did not affect the response patterns, but determined the identity of some of the populations stimulated. Most strikingly, the bacteria *Burkholderia*, *Lysobacter* and fungi *Rhizopus*, *Fusarium* were selectively stimulated. Given the ecological importance of these microbial groups as decomposers and/or plant pathogens, such regulation of the composition of microbial successions by soil history may have important consequences in terms of soil carbon turnover and crop health.

## Introduction

Soils are highly complex, heterogeneous matrices which shelter a huge diversity of organisms [1,2]. With thousands of different species hosted per gram of soil, microbial communities account for a large part of this biodiversity [3,4]. As a major component of the biosphere, soil also offers a support for plant and animal development, human activities, and is directly exposed to variations in climatic conditions. It is consequently a dynamic environment, and there is a considerable body of evidence that soil microbial diversity responds strongly to changes in soil conditions [5–7].

One key example is the pattern of microbial community dynamics induced by the addition of organic compounds such as plant debris into the soil [8–14]. Many studies have indeed reported a progressive and orderly pattern of community development, as regards the concept of species succession commonly employed to explain community dynamics in above-ground terrestrial plant communities [15]. Most of the community changes occur during the first month after the input, mainly attributed to fast-growing copiotrophic populations that take advantage of the readily degradable C-compounds (*i*.*e*. soluble compounds, cellulose, hemicellulose) in the plant residues [14,16,17]. Subsequently, more specialized slow-growing populations develop on the remaining recalcitrant C-compounds (*i*.*e*. lignin) due to their greater catabolic capacities compared to the early-stimulated populations [8,11,12,18]. Such patterns of diversity dynamics have been demonstrated for bacteria [13,14] and fungi [17]. In addition, since bacteria are assumed to harbor lower capacities for decomposing recalcitrant compounds than fungi [19], it is generally accepted that bacteria dominate the initial stages of decomposition whereas fungi dominate the later stages [20–22]. However, this is mainly based on quantitative estimates of bacterial and fungal communities. In contrast, studies addressing the response of the taxonomic composition of soil bacterial and fungal communities to plant residues addition are scarce and the relative contribution of bacterial *vs*. fungal diversity to the different stages of plant residues decomposition remains poorly documented [22,23].

In addition, while many studies have shown that land use management [24–28] as well as plant litter quality (*i*.*e*. initial C/N ratio, lignin content, etc…) [11,14,29] are important drivers of soil microbial diversity in a given soil, it is not known if the response of soil microbial communities to the addition of a fixed quality of plant residue depends on the soil’s history. This question is, however, of major importance since the intensity of the decomposition process and the resulting ecosystem services may depend on the diversity of the microbial populations responding to the input [14,30,31].

The aim of this study was to investigate the importance of soil management history in the response of the taxonomic diversity of bacterial and fungal communities to the incorporation of wheat straw. For this, a field experiment was set up at the long-term observatory of Lusignan (France). Briefly, the site was composed of two adjacent plots, each with a particular land use history (17 years grassland *vs*. 20 years cropland). Each plot was subdivided into 6 sub-plots, three of which were amended with wheat residues while the other three represented unamended controls. Following wheat addition, the dynamics of microbial diversity (fungal and bacterial) was followed in each sub-plot over a one-year period by high throughput sequencing of ribosomal genes. The site was kept vegetation-free throughout the experiment to avoid an additional response of microbial communities to plant development. To our knowledge this is the first study of the fine *in situ* characterization of the response of bacterial and fungal diversity to seasonal climatic fluctuations and plant residues addition in relation to the soil’s land use history.

## Materials and Methods

### Site description and sampling

The field experiment was set up on the long-term observatory for environmental research at Lusignan, France (SOERE-ACBB, http://www.soere-acbb.com/index.php/fr/). The soil on the site is a loamy textured Cambisol and the climate is oceanic with a mean annual temperature and precipitation of 10.5°C and 800 mm, respectively [32]. The experimental site was set up in two phases. First, in April 2011, the two main plots were established (S1 Fig). They were each 5×2 m in size and very close together (separated only by a 5 m pathway), but strongly contrasted in terms of land use history. One plot was set up on a soil that had been under crop rotation (wheat, barley and maize) for 20 years with annual tillage, herbicide crop treatment and nitrogen fertilization (ammonium nitrate: 100 kg/ha/year). The other plot was set up on permanent grassland (over 20 years old), fertilized (ammonium nitrate: 150 to 300 kg/ha/year) and harvested (3 to 4 times/year) for annual forage. The vicinity of the two plots was a prerequisite to ensure that differences in physicochemical and biological properties between the two plots were due to land use history, and not to spatial heterogeneity. Each plot (grassland and cropland) was weeded manually (shoots and roots) in order to remove the effect of plants on the soil microbial communities. After weeding, each plot was divided into six 0.7×0.7 m sub-plots (S1 Fig) and the site was left for 5 months to stabilize, with manual weeding every week to remove regrowth seedlings. In September 2011 the soil from each of the 6 sub-plots in each grassland and cropland plot was excavated to a depth of 10 cm. For each plot, soils from 3 of the sub-plots were amended homogeneously by mixing the excavated soil with wheat residues (250 g per sub-plots, corresponding to 5 t dry matter ha^-1^). Wheat residues originated from mature wheat plants (harvested 110 days after sowing) cultivated under controlled conditions (Groupe de Recherches Appliquées en Phytotechnologie, CEA Cadarache, France). The roots were separated from the shoots which were then oven-dried at 65°C for 48 h and cut to obtain straw residues 0.5 cm in length. The C/N ratio determined for wheat was 77.7 (elementar analyser Euro EA (EUROVECTOR, Milano, Italia)). The soil from the 3 other sub-plots was also mixed, but without addition of wheat straw. After mixing, the amended and non-amended soils were returned to their respective sub-plots. In this way, the site was finally composed of two plots, each representing a particular land use history (grassland *vs*. cropland), with two treatments per plot represented by triplicate wheat straw-amended sub-plots and triplicate non-amended control sub-plots.

To avoid horizontal transfers of organic matter between control and amended sub-plots, a protective tarp was buried to 30 cm depth around the control sub-plots. In addition, polyethylene safety nets with 10×10-mm diamond mesh were placed on the wheat straw-amended sub-plots to avoid migration of surface applied residues *via* runoff. Soil sampling was carried out regularly from September 2011 to September 2012, with more frequent sampling during the first month following wheat straw incorporation. This represented a total of 10 sampling dates: T0 (date of wheat straw addition); T3; T7; T22; T51; T85; T125; T211; T254; and T365 days, corresponding to 120 soil samples (3 replicates×2 treatments×2 plots×10 dates). After sampling, the soil was sieved to 2 mm, lyophilized and stored at -40°C before DNA extraction. In addition, part of the soil sampled at T0 was air dried for analysis of particle size distribution, pH, Soil Organic Carbon (SOC), Total Nitrogen (N-tot), soil C/N ratio, Soil Organic Matter (SOM), Phosphorous and Cation Exchange Capacity (CEC). Soil physicochemical properties were determined by the Soil Analysis Laboratory of INRA (ARRAS, France, http://www.lille.inra.fr/las).

### DNA extraction and molecular microbial biomass determination

Microbial DNA was extracted from 1 g of all 120 soil samples, using a single procedure standardized by the GenoSol platform (http://www.dijon.inra.fr/plateforme_genosol) [33]. DNA concentrations of crude extracts were determined by electrophoresis in 1% agarose gel using a calf thymus DNA standard curve, and used as estimates of microbial molecular biomass [34]. After quantification, DNA was purified using a MinElute gel extraction kit (Qiagen, Courtaboeuf, France).

### Pyrosequencing of 16S and 18S rRNA gene sequences

Bacterial and fungal diversities were determined at each sampling date, for each triplicate treatment (amended and control) of each land use history (grassland and cropland) by 454 pyrosequencing of ribosomal genes. For bacteria, a 16S rRNA gene fragment with sequence variability and the appropriate size (about 450 bases) for 454 pyrosequencing was amplified by PCR using primers F479 and R888. For fungi, an 18S rRNA gene fragment of about 350 bases was amplified using primers FR1 and FF390. Primers and PCR conditions were as described previously [7]. The PCR products were purified using a MinElute gel extraction kit (Qiagen, Courtaboeuf, France) and quantified using the PicoGreen staining Kit (Molecular Probes, Paris, France). A second PCR of 9 cycles was then conducted under similar PCR conditions with purified PCR products and ten base pair multiplex identifiers added to the primers at 5’ position to specifically identify each sample and avoid PCR bias. Finally, the PCR products were purified and quantified as previously described [7]. Pyrosequencing was then carried out on a GS FLX Titanium (Roche 454 Sequencing System).

### Bioinformatics analysis of 16S and 18S rRNA gene sequences

Bioinformatics analyses were done using the GnS-PIPE developed by the GenoSol platform (INRA, Dijon, France) and initially described by Terrat *et al*., [33]. First, all reads were sorted according to the chosen identifiers sequences. The raw reads were then filtered and deleted based on their: (a) length, (b) number of ambiguities (Ns), and (c) primer(s) sequence(s). PERL programs were then applied to obtain strict dereplication, alignment of reads using infernal alignments [35] and clustered at 95% sequence similarity into operational taxonomic units (OTU) that cluster rare reads with abundant ones, and do not count differences in homopolymer lengths. Another homemade filtering step was then applied to eliminate potential sources of errors (e.g., PCR chimeras, sequencing errors, OTU overestimation). In order to efficiently compare the datasets and avoid biased community comparisons, the samples reads were reduced by random selection closed to the lowest datasets (4,000 and 8,000 reads for 16S- and 18S-rRNA gene sequences respectively). The retained high-quality reads were used for: (*i*) taxonomy-independent analyses, to determine diversity indices using the defined OTU composition, and (*ii*) taxonomy-based analysis using similarity approaches against dedicated reference databases from SILVA (R114 for 16S, and R111 for 18S). The raw data sets are available on the EBI database system under project accession number [PRJEB6118].

### Statistical analysis

Differences in microbial communities structure between control and wheat-incorporated plots within a given land use history were characterized using UniFrac distances [36]. Non Metric Multidimensional Scaling (NMDS) was used to graphically depict differences between microbial communities. Analysis of SIMilarity (ANOSIM, 999 permutations) was used to test the significance of the observed clustering of samples on the ordination plot according to soil history. The difference in change of the microbial communities in response to wheat straw between the two land use histories was interpreted as the magnitude of the UniFrac distance between the control and the amended plots for each sampling date and was calculated as follows: magnitude of modifications for each sampling date and land use history = [Average of Inter UniFrac distances (control plots/ soil-incorporated plots)]–[((Average of Intra UniFrac distances control plots) + (Average of Intra UniFrac distances soil-incorporated plots))/2].

Heat maps were built up by applying the heatmap.2 function implemented in the gplots R package to the relative abundance values of the most dominant bacterial and fungal phyla across the samples (relative abundance > 1%). For the analysis, the datasets (soil histories) were analysed together, so the mean values of Z scores are from the total dataset. Each row was scaled so that the mean of each taxonomic group across sample types was calculated and colored according to the corresponding Z score. All these analyses were performed with the R free software (http://www.r-project.org/).

Physicochemical parameters, diversity metrics, Unifrac distance and relative abundance were compared between treatments and land use histories by two-way ANOVA and the differences between them by Fisher test (P<0.05). All these statistical calculations were carried out using XLSTAT software (Addinsoft).

## Results

### Soil physicochemical characteristics

Soil texture corresponded to a loamy sand soil (Table 1) and did not differ between the grassland and cropland plots. In contrast, soil chemical properties were slightly but significantly modified by land use history with higher values of SOM, SOC, N-Tot, Soil C/N and CEC in grassland soil, whereas pH and phosphorous content were higher in the cropland soil. No significant difference was observed between cropland and grassland soil for soil microbial biomass (Table 1).

**Table 1:** Soil physicochemical characteristics and soil microbial molecular biomass according to land use history at T0.

Parameters	units	Grassland	Cropland
*Physicochemical*			
Clay	g/kg	138 ± 3 [a]	130 ± 2 [b]
Fine silt	g/kg	367 ± 5 [a]	365 ± 2 [a]
Coarse silt	g/kg	321 ± 3 [a]	322 ± 3 [a]
Fine sand	g/kg	70 ± 7 [a]	75 ± 3 [a]
Coarse sand	g/kg	102 ± 7 [a]	107 ± 9 [a]
pH	-	6.02 ±.0.11 [a]	6.61 ±.0.05 [b]
SOC	g/kg	13.90 ± 0.41 [a]	10.63 ± 0.29 [b]
N-tot	g/kg	1.22 ±.0.03 [a]	0.98 ± 0.02 [b]
Soil C/N ratio	-	11.38 ± 0.10 [a]	10.85 ± 0.12 [b]
SOM	g/kg	24.05 ± 0.69 [a]	18.42 ± 0.49 [b]
Phosphorous	g/kg	0.09 ± 0.00 [a]	0.10 ± 0.00 [b]
CEC	cmol+/Kg	6.99 ± 0.25 [a]	6.30 ± 0.08 [b]
*Biological*			
SMB	μg NA/g	34 ± 5.8 [a]	38 ± 6 [a]

Abbreviations: SOC: Soil Organic Carbon, N-tot: soil total Nitrogen, SOM: Soil Organic Matter, CEC: Cation Exchange Capacity, SMB: Soil Microbial molecular Biomass.

Values are means (n = 6) ± standard errors.

Letters in brackets indicate significant differences between cropland and grassland, according to *Fisher* test (P<0.05)

### Impact of land use history and seasonal climatic variations on soil microbial diversity

**Fig 1:**
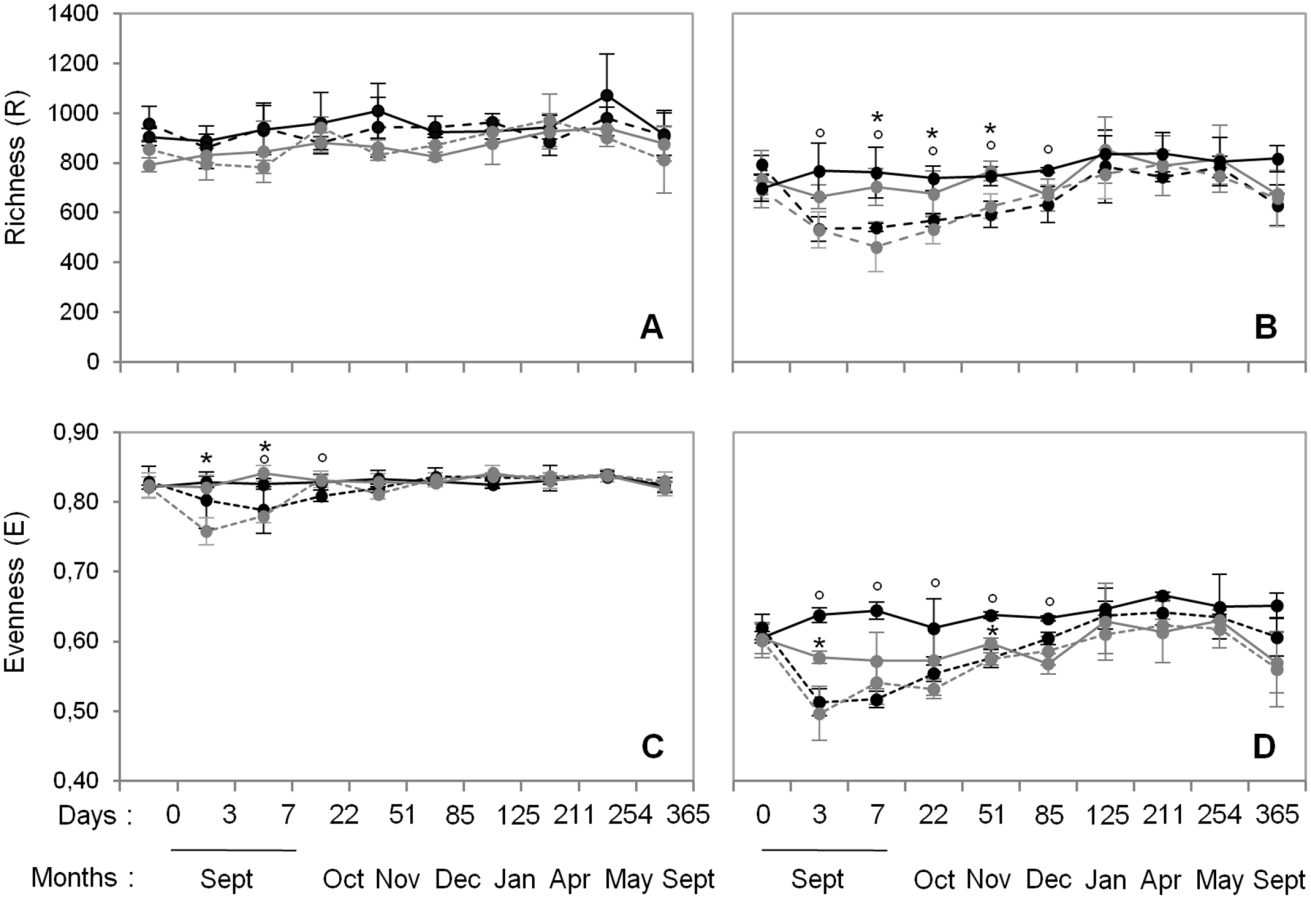
Dynamics of bacterial (A and C) and fungal (B and D) richness and evenness during the 12-month experiment in control (full line) and wheat-amended plots (dashed line) for each soil history: grassland (grey) and cropland (black). Error bars denote standard deviation of biological replicates (n = 3). Symbols in superscript indicate significant differences according to *Fisher* test (P<0.05) between control and corresponding wheat amended sub-plots for each grassland (stars) and cropland (circles) history.

Pyrosequencing yielded a total of 1,844,885 and 2,020,216 sequences of 16S- and 18S-rDNA with 4000 and 8000 quality sequences per sample, respectively. Diversity indices calculated from the sequence datasets obtained from control plots (without wheat addition) showed that, overall, both bacterial and fungal richnesses were higher in cropland plots than in grassland plots whatever the sampling dates (Fig 1). Contrastingly, bacterial evenness was similar in the two soils whereas fungal evenness was lower in grassland than in cropland soil. Interestingly, bacterial and fungal diversity indices did not vary significantly during the 12-month experiment whatever the soil history.

**Fig 2:**
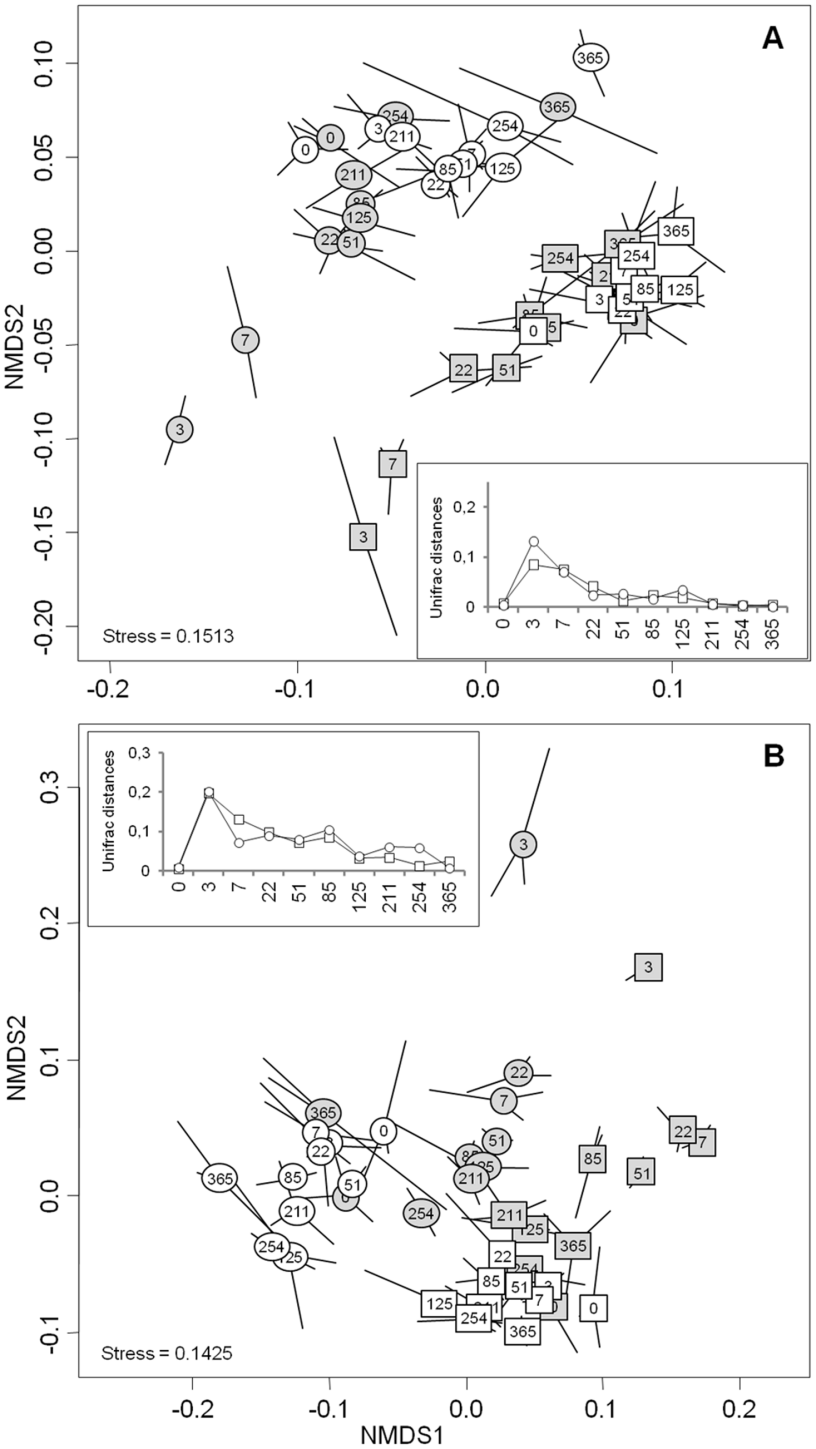
Non-metric multi-dimensional scaling (NMDS) ordination plot derived from weighted pairwise Unifrac distances for (A) bacterial and (B) fungal communities over time according to soil management history: cropland (squares) and grassland (circles). White labels represent the control treatment and grey labels represent the wheat-amended treatment. Numbers represent sampling dates and lines correspond to the three replicate samples obtained at each sampling date. Stress values for the two ordination plots were < 0.2 which indicates that these data were well-represented by the two dimensional representation. *inset* represents magnitude changes in Unifrac distance between the control and amended sub-plots over the year.

NMDS analysis of the full bacterial- (Fig 2A) and fungal-sequences datasets (Fig 2B) highlighted distinct microbial community structure between the control subplots depending on the land use history (confirmed by Pairwise ANOSIM test (R = 0.805, P = 0.001 for bacteria and R = 0.916, P = 0.001 for fungi)). A difference between the two soils was also observed at the OTU level, with only 21.4% of bacterial and fungal OTU being shared at T0 (data not shown). The taxonomic affiliation of 16S- and 18S-rDNA sequences at T0 showed that overall the microbial phyla distribution within these two soils was dominated by *Proteobacteria*, followed by *Actinobacteria*, *Firmicutes*, *Acidobacteria*, *Bacteroidetes*, and *Chloroflexi* for bacteria, and by *Basidiomycota* and *Ascomycota* for fungi. However, the difference between the two soils was mainly due to an increase in the relative proportions of *Proteobacteria* (58.6 to 54.7%), *Actinobacteria* (11.8 to 8.5%), *Acidobacteria* (8.9 to 7.8%), and *Basidiomycota* (45.3 to 21.2%) in grassland, as compared to cropland soil which harbored higher relative proportions of *Gemmatimonadetes* (2.4 to 1.2%), *Chloroflexi* (4.5 to 1.7%), *Nitrospirae* (1.3 to 0.6%), and *Ascomycota* (55.7% to 29%).

**Fig 3:**
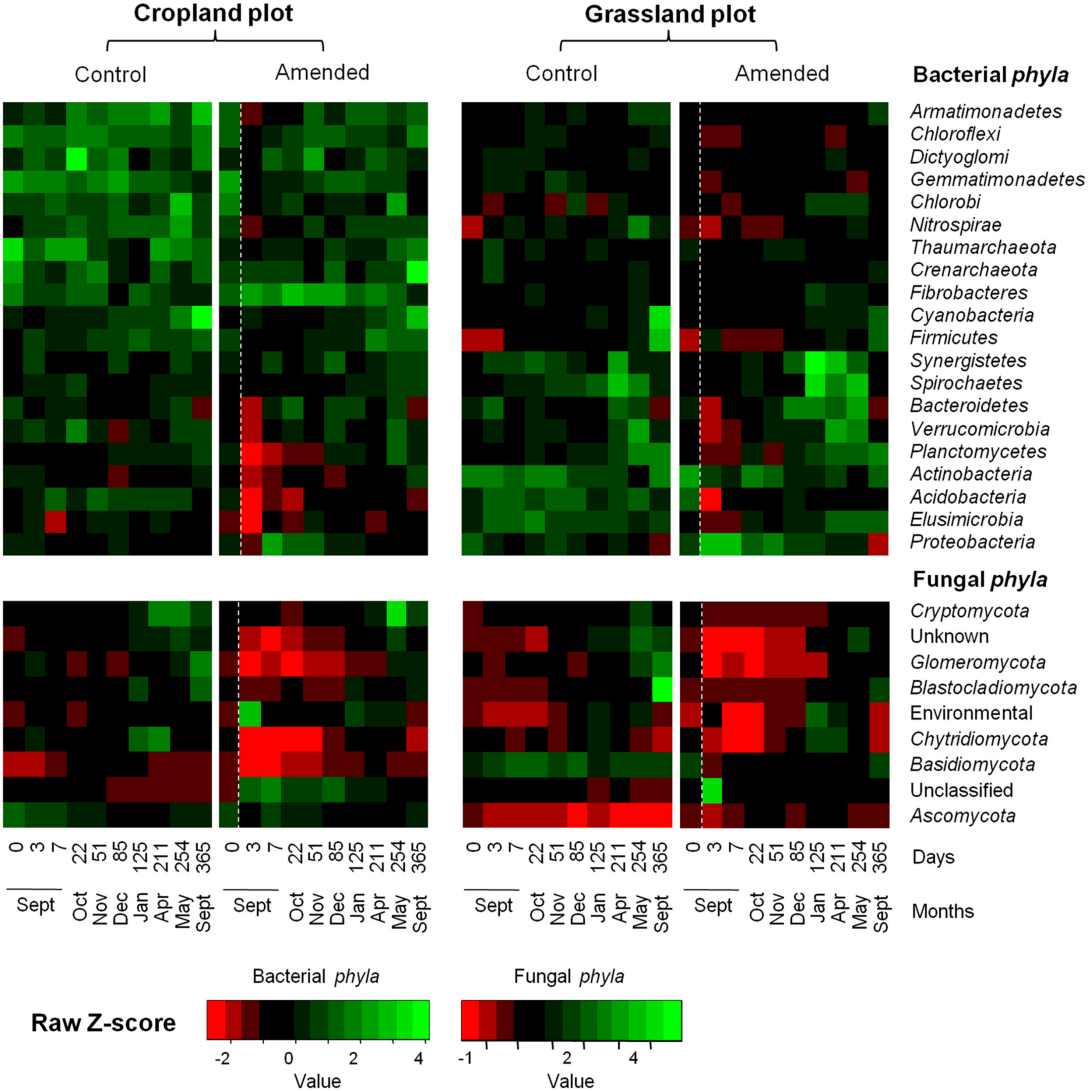
Heat map comparison of bacterial and fungal phyla detected in soils according to land management history (cropland and grassland) and treatments (control or amended) over time. In the amended treatment, the white dashed line represents the time of wheat straw input. The captions show the Z-scores (relative abundances are expressed as median centered Z-scores between all samples, and the colors are scaled to the standard deviations. For each sampling date, the average relative abundance was obtained from the biological replicates (n = 3).

As observed for the diversity indices, bacterial and fungal structures (Fig 2) in the controls did not change significantly during the experiment, with all sampling dates being grouped together on the factorial map. This stability of the microbial communities was also observed when the bacterial taxonomic composition was analysed at both phylum (Fig 3) and genus levels (S2 and S3 Figs).

### Response of soil microbial diversity to wheat residues according to land use history

Wheat residues addition led to strong modifications of soil microbial diversity whatever the soil history (Fig 1). All microbial diversity indices (bacterial and fungal) were decreased during the first week following straw incorporation (Fig 1), except for bacterial richness, which was similar in both control and amended treatments irrespective of soil history (Fig 1A). Following this first phase of decline, the indices then increased and were resilient, stabilizing at the level of the respective controls. However, resilience occurred more rapidly for bacteria (22 days for bacterial evenness) than for fungi (85 days for both fungal richness and evenness). In addition, the overall amplitude of the modifications in diversity indices was greater for fungi than for bacteria.

Microbial community structure also responded strongly to wheat addition (Fig 2). Similar patterns of community dynamics were observed for bacteria and fungi whatever the soil history, characterized by a highly dynamic phase during the first week after wheat addition followed by a slowdown and resilience of community modification. The community changes peaked after 3 days for both bacteria and fungi whatever the soil history (Fig 2), as observed for the diversity indices. However, the amplitude of the structural modifications was greater for fungi than for bacteria, and resilience of the community structure occurred more rapidly for bacteria (51 days) than for fungi (365 days), although the time of occurrence did not depend on the land use history (Fig 2).

**Fig 4:**
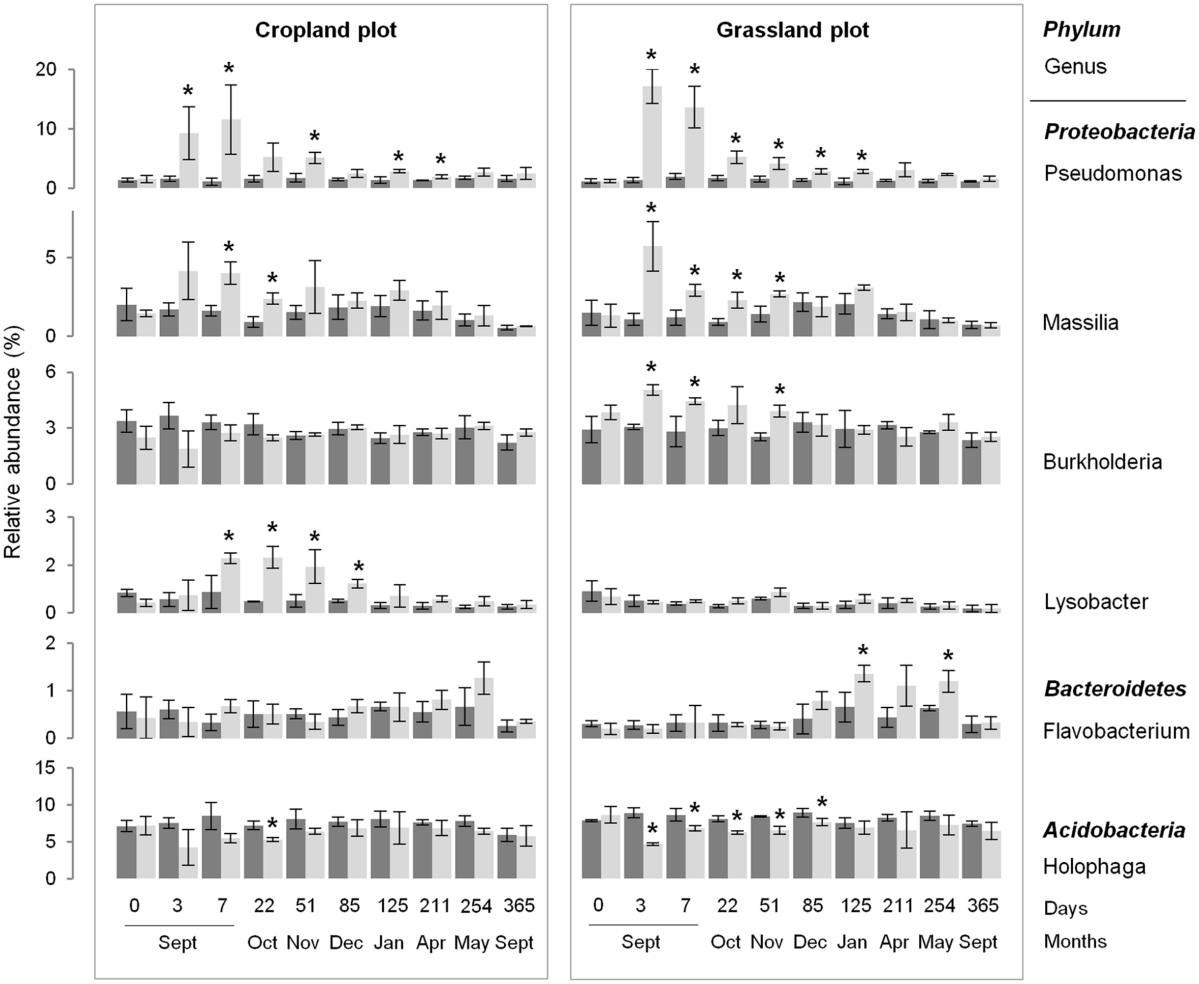
Dynamics of the relative abundance of some bacterial genera over time, according to soil management history (cropland and grassland) and treatment: control (dark grey) and wheat-amended (light grey). Error bars denote standard deviation of biological replicates (n = 3). Asterisks indicate significant difference between control and amended treatments at each sampling date, according to *Fisher* test (P<0.05).

**Fig 5:**
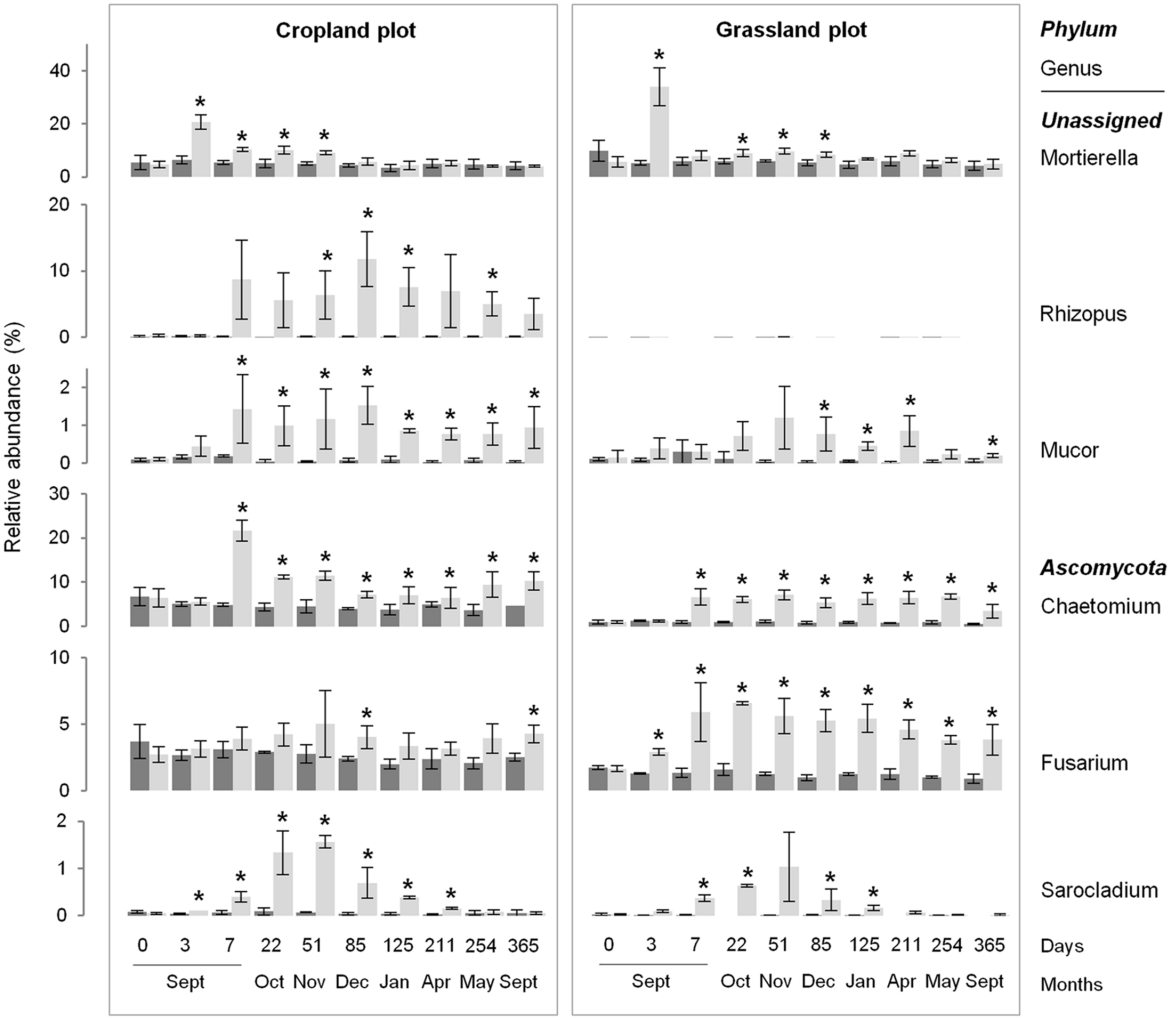
Dynamics of the relative abundance of some fungal genera over time, according to soil management history (cropland and grassland) and treatment: control (dark grey) and wheat-amended (light grey). Error bars denote standard deviation of biological replicates (n = 3). Asterisks indicate a significant difference between control and amended sub-plots at different sampling dates, according to *Fisher* test (P<0.05).

Despite the similar community response patterns (Figs 1 and 2), the community structures (bacterial and fungal) at each sampling date could still be clearly discriminated between the two soils (grassland *vs*. cropland), indicating that different communities were stimulated by wheat residues (Fig 2). This observation was confirmed by the taxonomic compositions at the phyla and genera levels (Fig 3; S2 and S3 Figs). Some microbial phyla responded similarly to wheat addition (Fig 3) in the two soils. Thus, *Proteobacteria* and the group of sequences “unclassified” to fungal phyla were both strongly increased during the early stage following residues input. Other bacterial phyla, such as *Nitrospira*, *Verrumicrobia*, *Acidobacteria*, *Actinobacteria*, and *Planctomycetes* were decreased, as well as the fungal phyla *Basidiomycota*, *Chytridiomycota*, and *Glomeromycota*. In addition to these “shared response” groups, some phyla responded specifically according to the soil history. For example, the early increase of *Fibrobacteres* (from 3 to 125 days following wheat addition) occurred only in the cropland soil whereas *Synergistetes* and *Bacteroidetes* increased much later (from 85 to 254 days following wheat addition) and only in grassland soil. In the same way, *Ascomycota* increased mainly in the grassland soil throughout the experiment. Analysis of taxonomic composition at the genera level provided complementary information to the phyla data. For instance, the general increase in *Proteobacteria* was mainly explained by the stimulation of *Pseudomonas* and *Massilia*, whereas *Lysobacter* only increased in the cropland soil, and *Burkholderia* only increased in grassland soil (Fig 4). Similarly, the group of sequences “unclassified” to fungal phyla, which represented the principal group of stimulated fungal sequences regardless of soil history, mainly consisted of populations affiliated to *Mucor*, for which the relative abundance increased throughout the experiment; *Mortierella*, which also increased in both soils, but just from day 0 to day 3; and *Rhizopus*, which increased strongly in cropped soil but not in grassland soil (Fig 5).

## Discussion

Bacterial and fungal diversity and composition differed according to the soil management history (grassland *vs*. cropland), as reported in previous studies [26,37]. In addition, discrimination between the two soils remained stable throughout the one-year experiment, highlighting the long-lasting nature of the changes induced by past land use [26,38,39]. Since the site was kept weed-free, it is likely that the differences in soil properties accounted for a large part of the microbial communities discrimination between the two soils [40–43]. While soil texture was similar, as expected because of the vicinity of the two plots, soil chemical properties differed between the grassland and cropland soils. Thus SOC and soil nitrogen content were higher, and pH lower in the grassland soil, in agreement with other studies [39,44]. Accordingly, microbial groups previously described as mainly copiotrophic, such as *Proteobacteria* [14,18], or acidophilic, such as *Acidobacteria* [45,46]were more represented in the grassland soil than in the cropland soil where oligotrophic groups such as *Gemmatimonadetes* were more represented [16,47]. In addition, *Ascomycota*, one of the most dominant fungal groups in cultivated soils [48,49], dominated the fungal community in the cropland soil. Contrastingly, the abundance of *Basidiomycota* and *Actinobacteria*, both described as k-strategists, being favored in more stable systems such as forest [50] and natural grassland [24,51,52], was increased in the grassland soil.

In the controls, neither bacterial nor fungal diversity (*i*.*e*. richness and evenness) nor composition varied significantly during the one-year period despite the climatic variations that occurred at the experimental site (S4 Fig). This contrasted with Lauber and colleagues [6] who evidenced a significant *in situ* temporal variability of bacterial communities over a 7-month period. These authors showed however that a large proportion of the seasonal variations in bacterial diversity could be explained by the diversity and dynamics of plant cover and the subsequent regulation of C-inputs (*i*.*e*. root exudates and plant litter). Thus, the observed stability of microbial diversity in our study might be explained by the exclusion of plants. Interestingly, this suggests that under a buffered oceanic climate, as is the case at our experimental site, the dynamics of plant cover rather than climatic variations may shift microbial diversity during the seasons.

Soil microbial diversity responded strongly to wheat straw incorporation, in agreement with many other studies focused on the impact of plant litter on soil microbial communities [8,9,12,13,17]. Microbial richness and evenness indices showed a transient decrease following amendment, due to the early stimulation of a subset of fast growing bacterial and fungal populations developing on the most easily decomposable straw-compounds [14,17,22]. This was in agreement with the patterns of community structure, that were characterized by an initial early and highly dynamic phase followed by a second phase of slower variations leading to a resilience and stability of both bacterial and fungal communities structure. It is noteworthy that for a given community (*i*.*e*. bacteria or fungi), in spite of initial differences in terms of diversity and composition, neither the variations in diversity indices nor the patterns of community structure differed according to the soil management history. However, the kinetics and amplitude of community response did differ between bacteria and fungi. Surprisingly, the peak in communities’ modification, for both bacteria and fungi, occurred three days after the input, indicating that fungi may be as competitive as bacteria for fast development on freshly incorporated wheat straw. This is not in agreement with the commonly accepted idea that differences in physiology and metabolic capacities between bacteria and fungi may confer an advantage to bacteria over fungi during the early stages of decomposition [19,21,53]. However, recent studies using ^13^C labelling of plants also showed that decomposer fungi may contribute significantly to the decomposition of simple root exudates [54,55]. In addition, the quality of the wheat straw, characterized by a high C/N (77.7), may also have contributed to the concomitant, early stimulation of bacterial and fungal populations, as well as the greater amplitude of the diversity modification observed for fungi compared to bacteria. Indeed, fungi are known to be better equipped than bacteria for decomposing recalcitrant organic C-compounds [20–22] and the decomposability of the litter entering the soil is likely to be of major importance in determining the sequential stimulation of bacteria and fungi during the early phase of decomposition. In other respects, the better enzymatic abilities of fungal populations may explain why fungal communities responded up to 122 days whereas modifications of the bacterial communities occurred only during the first month (22 days) after wheat addition. This slower resilience of fungal diversity, as compared to that of bacteria, suggests that fungi may dominate the latter stages of wheat straw decomposition [19,21].

While the patterns of bacterial and fungal diversity indices and communities structure were similar, regardless of soil history, some of the populations entering the successions differed. Several bacterial phyla, such as *Nitrospira*, *Verrumicrobia*, *Acidobacteria* and *Planctomycetes*, were decreased by wheat straw incorporation, which was in agreement with other studies showing that these phyla are probably mainly composed of oligotrophic slow-growing populations [14,16,18]. In addition, *Nitrospira* underrepresentation might also be response of reduction of nitrogen availability due to high C/N material input. Contrastingly, *Bacteroidetes* were stimulated by wheat straw, but in the latter stages of wheat decomposition (*i*.*e*. 5 months after wheat addition), in agreement with previous results [29]. The *Proteobacteria phylum* was increased rapidly regardless of the soil history, as reported previously [29,56]. This global increase was mainly due to the stimulation of two genera occurring in both soils: *Pseudomonas* and *Massilia* that had previously been described as mainly copiotrophic bacteria being stimulated by inputs of wheat straw or more soluble C-compounds [16,57,58]. Interestingly, in addition to these genera, *Burkholderia* was enhanced only in the soil with a grassland history whereas *Lysobacter* was enhanced only in the soil with a cropland history. Recent studies also reported stimulation of *Burkholderia* species by plant residue inputs into the soil [23], probably because of the strong catabolic versatility of these species which enables them to degrade a wide range of C-compounds including cellulose [59]. Since this *genus* is also known to be closely associated with plants in the rhizosphere [23], it is likely that the long grassland history, characterized by permanent plant cover with a very dense root network, may have led to the selection of a particular diversity of *Burkholderia* species, compared to the adjacent cropland, hence explaining the observed discrepancy between the responses of the two soils. However, differential stimulation of this *genus* may be of significance for subsequent crops since many *Burkholderia* species are associated with plants in positive (*i*.*e*. growth promoting species) or negative (*i*.*e*. pathogens) interactions [59]. Similarly to *Burkholderia*, *Lysobacter* is known to possess strong lytic abilities to degrade various biomacromolecules [60]. However, this *genus* has also been shown to decrease with decreasing soil pH [60], which may explain why stimulation was observed in the cropland soil (pH 6.6), but not in the grassland soil (pH 6.0). Altogether, these results suggest that the modulation in diversity of the populations responding to plant litter input, according to the past history of the soil, may be determined either by the actual soil properties or by the diversity of the decomposers resulting from past interactions with plant cover.

Regarding the fungal communities, the observed initial decrease of the diversity indices was due to the massive increase of *Mortierella*, which represented up to 20 and 30% of the sequences identified at T3 in the cropland and grassland soils, respectively. This strong stimulation, observed only three days after wheat straw incorporation, suggests that this *genus* may be as competitive as bacteria in decomposing the most decomposable straw C-compounds. This is in agreement with other reports that *Mortierella* are fast growing fungi developing on substrates rich in simple carbohydrates [61,62]. In addition, their ability to produce chitinolytic enzymes may make them successful competitors among other fungi [61], which could explain their massive and exclusive development observed at T3. Nevertheless, this competitive advantage may be limited to decomposition of the most soluble wheat compounds since *Mortierella* decreased from T7 and was replaced by a diversity of other newly stimulated fungi. As for bacteria, some of these new populations were shared or specifically stimulated depending on the soil history. For example, *Mucor* and *Chaetomium* were stimulated in both soils from T7 and throughout the year of experimentation, in agreement with their known contribution to plant debris decomposition in soil due to their cellulolytic, hemicellulolytic and lignolytic abilities [63,64]. The *genus Sarocladium* was also stimulated in both soils, but only transiently, with a peak observed at T51. Interestingly, two fungal genera responded specifically depending on the soil history. Most strikingly, *Rhizopus* was very weakly represented in the controls of both soils, but responded strongly and lastingly to wheat straw incorporation in the cropland soil (representing up to 15% of the sequences identified) but not in the grassland soil. This *genus* is a common soil mold which develops on decomposing plant litter in soil [62]. However, we cannot explain why it only responded in the cropland soil. It is possible that this selective stimulation could be of significance for the health of subsequent crops since some *Rhizopus* species are described as plant pathogens [61]. In contrast to *Rhizopus*, *Fusarium* was one of the dominant fungi in both soils and was strongly stimulated in the grassland soil but not in the cropland soil. This *genus* is known be saprophytic, widely distributed in soil and often associated with plant debris [65]. A similar stimulation of *Fusarium* populations following crop residues incorporation, and the strong influence of the residues composition on the structure of the *Fusarium* community, has already been reported [66]. Here, we show that the stimulation of *Fusarium* by plant residues may also depend on the past use of the soil. In addition to potential repercussions in terms of carbon mineralization, this differential stimulation between the two soils may have consequences for subsequent crops since some of the *Fusarium* species are strongly associated with several diseases that cause crop yield losses and mycotoxin contamination of grain [65]. However, since all *Fusarium* species are not pathogenic, additional determination of *Fusarium* diversity at the species level would now be needed to determine whether populations differentially stimulated were pathogenic or not.

This study provides new insights into the *in situ* dynamics of the diversity of bacterial and fungal communities in soil. It shows that seasonal fluctuations of climatic conditions may have little impact on the diversity of these communities. While long-term differences in land use management impacted soil microbial diversity, they did not affect the patterns of community response to wheat straw incorporation in terms of diversity indices and community structure variations. Fungal diversity responded as quickly as bacterial diversity but the resilience of fungal communities lasted longer, which would indicate that whereas bacteria and fungi may both play a major role during the initial stages, fungi may dominate the latter stages of wheat decomposition. Despite similar response patterns, the identity of some of the bacterial and fungal populations in the successions was dependent on soil history. Among the specifically stimulated populations were bacterial and fungal populations of particular ecological importance, such as *Burkholderia* or *Fusarium*, both recognized for their involvement in the decomposition of organic matter, but also for their impact on crops health. This effect of soil history on population successions during the decomposition of a given quality of plant litter had not been evidenced previously. It is however of major importance since it highlights that the diversity of microbial decomposers may be driven by soil management history, with potential consequences in terms of plant residues decomposition, a transformation of significance for the C-balance in the soil environment.

## Supporting Information

S1 Fig Organization of the experimental site set up at the SOERE-ACBB (Lusignan, France). Pictures of the cropland (**A**), and grassland plot (**B**) associated with their respective diagrammatic representations (C and D). The two plots (2.5 × 5 m) were separated by a 5 meters pathway. Each plot was divided into 6 subplots of 0.49 m² (0.7 × 0.7), with 3 subplots amended with wheat straw (dotted line), and 3 unamended control subplots (full line). Grey squares in each subplot correspond to the fix part of gaz sampling chambers.
(TIF)

S2 Fig Heat map comparison of bacterial genera (relative abundance > 1%) detected in soils according to land management history (cropland and grassland) and treatments (control or amended) over time. In the amended treatment, the white dashed line represents the time of wheat straw input. The legend shows the Z-scores (relative abundances are expressed as median centered Z-scores between all samples, and the colors scaled to standard deviations). For each sampling date, the average of relative abundance was based on the biological replicates (n = 3).
(TIF)

S3 Fig Heat map comparison of fungal genera (relative abundance > 1%) detected in soils according to land management history (cropland and grassland) and treatments (control or amended) over time. In the amended treatment, the white dashed line represents the time of wheat straw input. The legend shows the Z-scores (relative abundances are expressed as median centered Z-scores between all samples, and the colors scaled to standard deviations). For each sampling date, the average of relative abundance was based on the biological replicates (n = 3).
(TIF)

S4 Fig Changes in the climatic parameters at the site during the 1-year period (01/09/2011 to 13/09/2012). Precipitation (A), soil humidity (black line) and temperature (grey line) (B). All these data were provided by the SOERE-ACBB (http://www.soere-acbb.com/index.php/fr/). Signs (×) indicate time points when soils were sampled for the biological molecular analysis.
(TIF)
