# Supplementary figures and images for: Land Use History Shifts In Situ Fungal and Bacterial Successions following Wheat Straw Input into the Soil
Source: PLoS One. 2015 Jun 23;10(6):e0130672. doi: 10.1371/journal.pone.0130672 (PMC4478037; doi:10.1371/journal.pone.0130672)

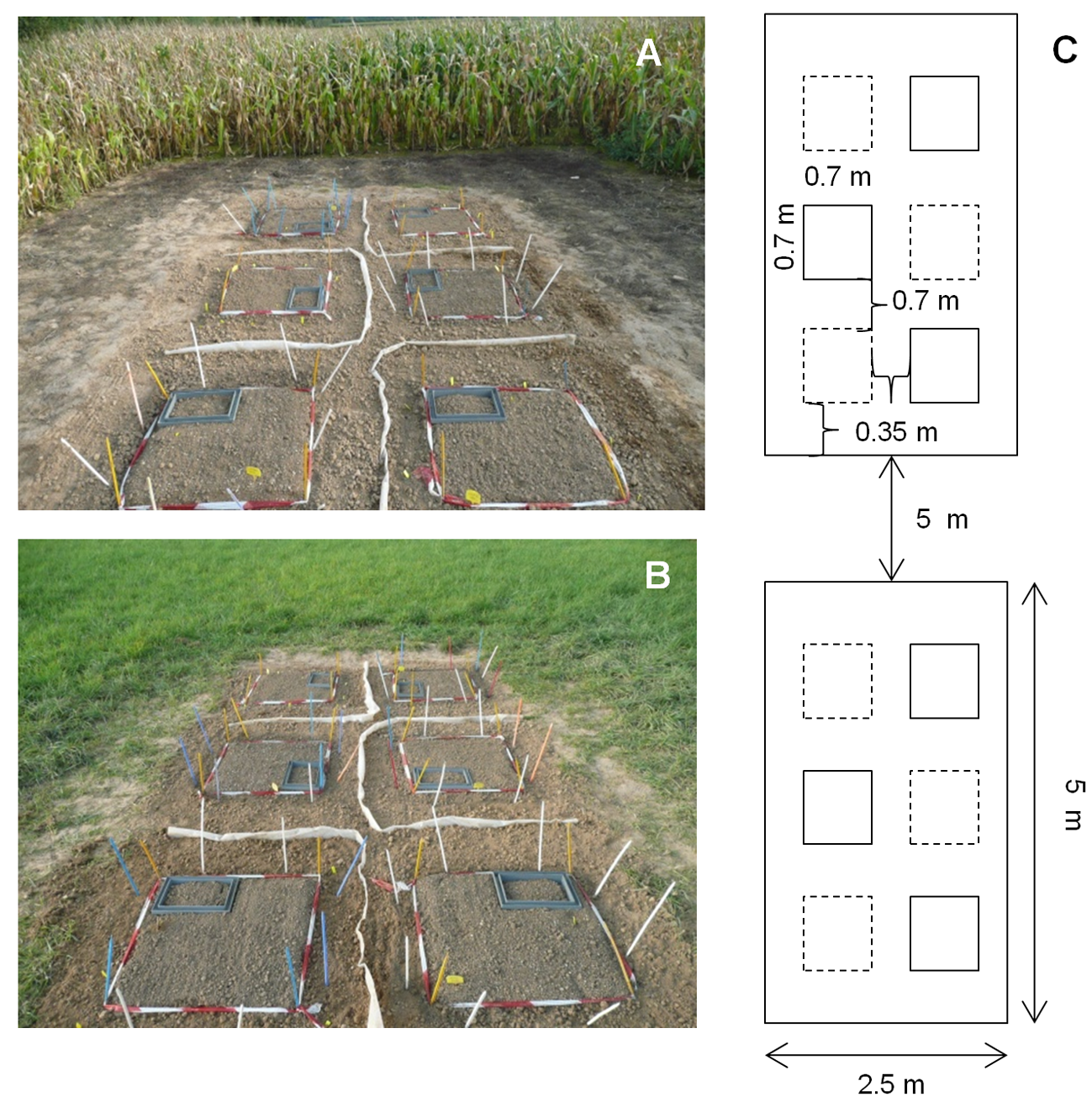

Supplement: S1 Fig — Pictures of the cropland (A), and grassland plot (B) associated with their respective diagrammatic representations (C and D). The two plots (2.5 × 5 m) were separated by a 5 meters pathway. Each plot was divided into 6 subplots of 0.49 m² (0.7 × 0.7), with 3 subplots amended with wheat straw (dotted line), and 3 unamended control subplots (full line). Grey squares in each subplot correspond to the fix part of gaz sampling chambers. (TIF) [file pone.0130672.s001.tif]

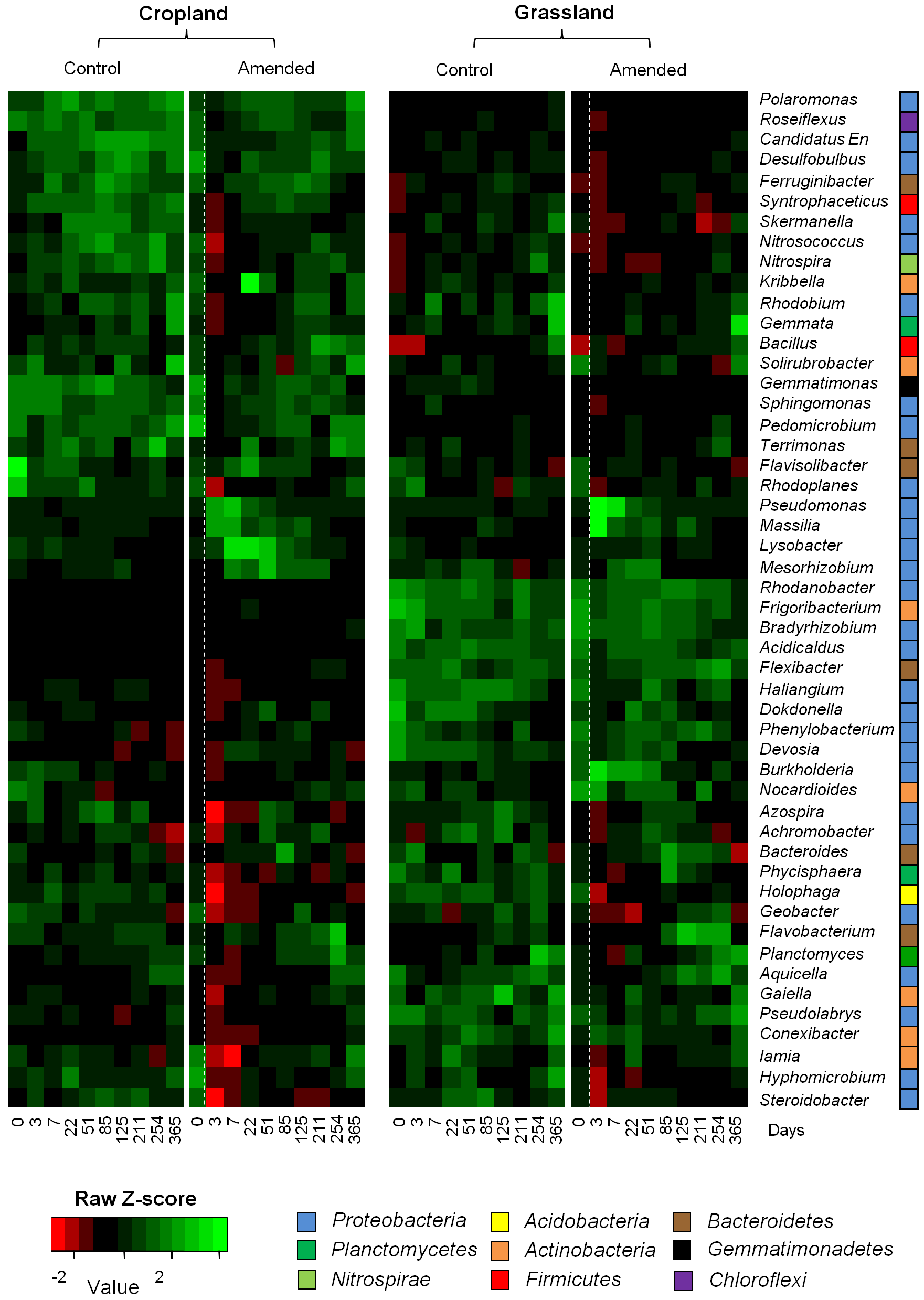

Supplement: S2 Fig — In the amended treatment, the white dashed line represents the time of wheat straw input. The legend shows the Z-scores (relative abundances are expressed as median centered Z-scores between all samples, and the colors scaled to standard deviations). For each sampling date, the average of relative abundance was based on the biological replicates (n = 3). (TIF) [file pone.0130672.s002.tif]

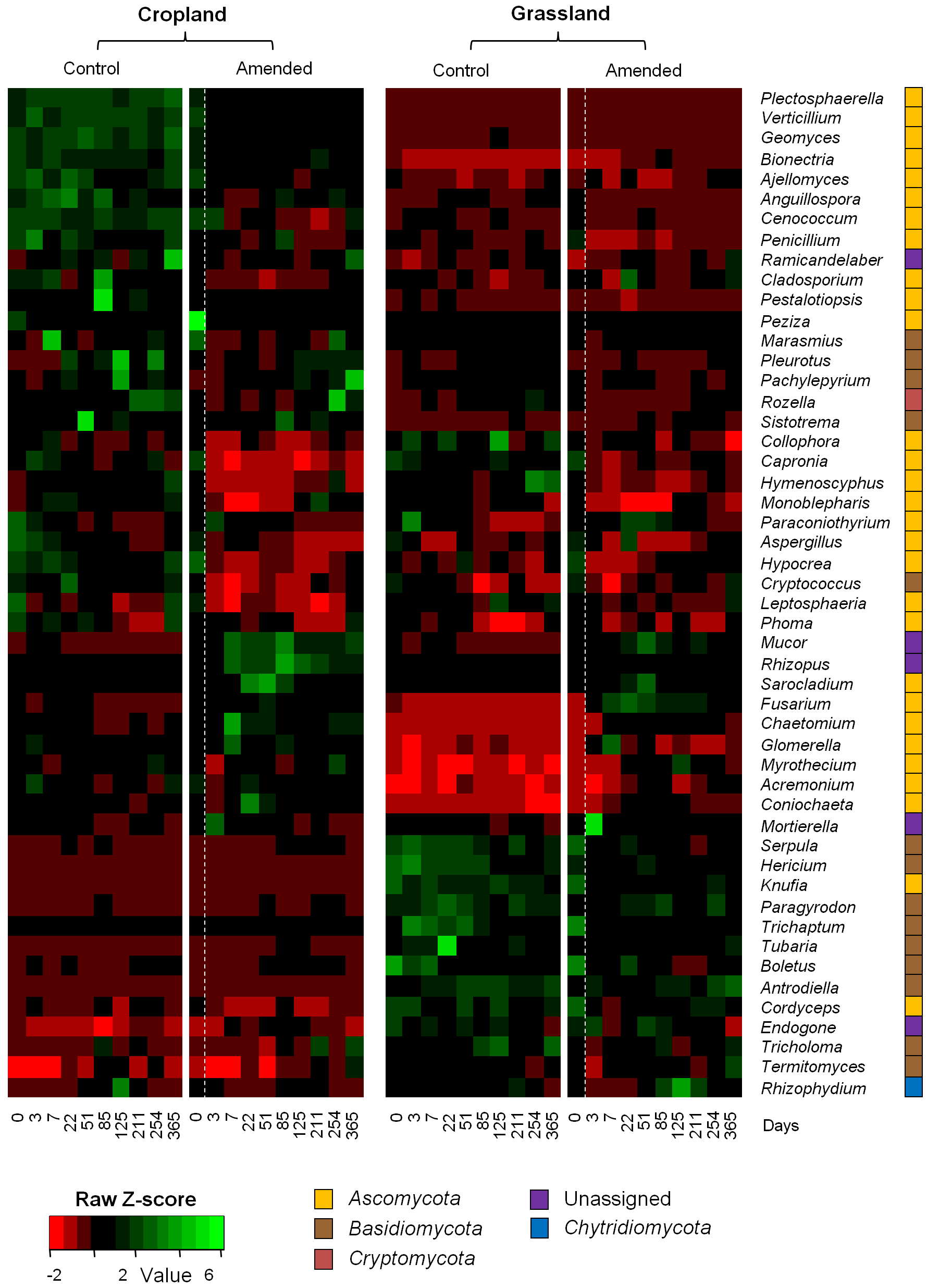

Supplement: S3 Fig — In the amended treatment, the white dashed line represents the time of wheat straw input. The legend shows the Z-scores (relative abundances are expressed as median centered Z-scores between all samples, and the colors scaled to standard deviations). For each sampling date, the average of relative abundance was based on the biological replicates (n = 3). (TIF) [file pone.0130672.s003.tif]

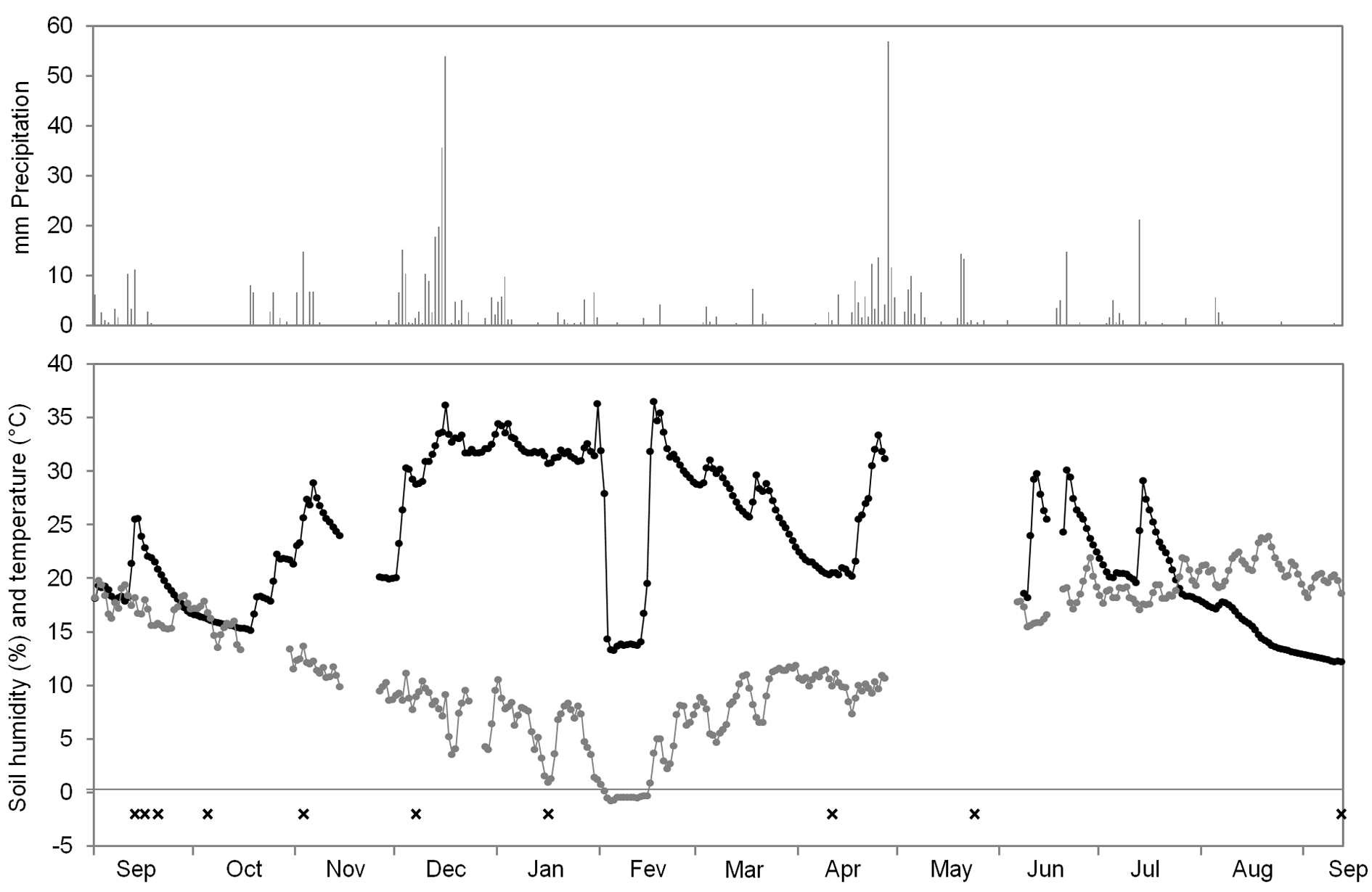

Supplement: S4 Fig — Precipitation (A), soil humidity (black line) and temperature (grey line) (B). All these data were provided by the SOERE-ACBB (http://www.soere-acbb.com/index.php/fr/). Signs (×) indicate time points when soils were sampled for the biological molecular analysis. (TIF) [file pone.0130672.s004.tif]
